# Clinical outcomes of personalized blastocyst embryo transfer after endometrial receptivity analysis: A multicenter, retrospective cohort study

**DOI:** 10.1002/rmb2.12550

**Published:** 2023-11-29

**Authors:** Yuya Takeshige, Seung Chik Jwa, Yasushi Hirota, Yutaka Osuga, Takeshi Kuramoto, Yasuyuki Mio, Kenji Furui, Masayuki Kinutani, Masahide Shiotani, Yoshimasa Asada, Hirobumi Kamiya, Hiroaki Yoshida, Hideki Igarashi, Koichi Kyono

**Affiliations:** ^1^ Kyono ART Clinic Takanawa Tokyo Japan; ^2^ Department of Obstetrics and Gynecology Saitama Medical University Saitama Japan; ^3^ Department of Obstetrics and Gynecology Jichi Medical University Tochigi Japan; ^4^ The University of Tokyo Tokyo Japan; ^5^ Kuramoto Women's Clinic Fukuoka Japan; ^6^ Mio Fertility Clinic Tottori Japan; ^7^ Clinic Mama Gifu Japan; ^8^ Kinutani Women's Clinic Hiroshima Japan; ^9^ Hanabusa Women's Clinic Kobe Japan; ^10^ Asada Ladies Clinic Nagoya Japan; ^11^ Kamiya Ladies Clinic Nagoya Japan; ^12^ Sendai ART Clinic Sendai Japan; ^13^ Kyono ART Clinic Sendai Sendai Japan

**Keywords:** age distribution, assisted reproductive technique, embryo implantation, embryo transfer, infertility

## Abstract

**Purpose:**

To evaluate clinical outcomes after endometrial receptivity analysis (ERA).

**Methods:**

This was a multicenter, retrospective cohort study involving 861 women who underwent ERA testing at certified fertility clinics in Japan, and who received subsequent personalized blastocyst embryo transfers (ET) between 2018 and 2020. Clinical outcomes, including pregnancies, miscarriages, and live births, were evaluated according to receptivity status for ERA.

**Results:**

Mean patient age was 37.7 years (SD = 4.0), and the median number of previous ETs was 2 (interquartile range, 2–3). 41.0% (353/861) of patients were non‐receptive for ERA testing. Clinical pregnancy, miscarriage, and live birth rates for personalized blastocyst ET were 44.5% (226/508), 26.1% (59/226), and 26.8% (136/508) for receptive patients, and 43.1% (152/353), 28.3% (43/152), and 28.9% (102/353) for non‐receptive patients, all statistically nonsignificant. Multiple logistic regression demonstrated similar nonsignificant associations between receptivity and clinical outcomes. Greater patient age, smoking, and longer duration of infertility were significantly and negatively associated with receptivity, whereas a history of delivery was positively associated and statistically significant.

**Conclusions:**

Clinical outcomes after ERA testing were similar between receptive and non‐receptive patients. Further prospective study including an appropriate comparison group are warranted to evaluate the efficacy of ERA testing.

## Graphical abstract

This paper discusses the effectiveness of ERA testing based on ERA results and clinical outcomes. A retrospective study with the largest number of patients has been conducted and is expected to provide useful findings.

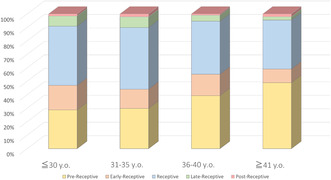

## 1 INTRODUCTION

Synchronization of a competent embryo and uterine endometrium are essential for successful embryonic implantation and pregnancy in humans.^1^ Decidual transformation of the endometrium is initiated by postovulatory elevation of progesterone levels in order to adapt to invasive and chromosomally diverse embryos, whereas that process is initiated by embryo implantation in most other mammals.^2^ Decidual transformation of the uterine endometrium initiates an acute inflammatory reaction, including immune cells, cytokines, and growth factors, coinciding with endometrial receptivity in what is known as the window of implantation (WOI).^3^ Impaired decidualization of the endometrium is associated with implantation failure and subsequent pregnancy complications, including recurrent miscarriages^4^, ^5^ and preeclampsia.^6^, ^7^

Endometrial receptivity analysis (ERA) was first introduced in 2011 to evaluate the expression levels of 238 endometrial receptivity‐related genes in human pre‐decidualized and decidualized endometria using microarray technology.^8^ Using a specific prediction algorithm, and based on expression analysis of those genes, this technology can identify an optimal WOI with high accuracy and reproducibility.^9^

To date, however, benefits and clinical efficacy of ERA testing and subsequent personalized embryo transfer (ET) remain a matter of debate.^8^, ^10^, ^11^, ^12^, ^13^ Many previous studies suffered from limited sample size.^12^, ^13^ Further, even though various factors such as recurrent implantation failure (RIF) and chronic endometritis could affect ERA test results,^8^, ^14^ no studies have specifically investigated whether patient characteristics and treatment factors are associated with receptivity. Thus, the current study investigated clinical outcomes of personalized blastocyst ET after ERA testing and factors affecting receptivity involving 861 women who received ERA testing at certified fertility clinics in Japan.

## 2 MATERIALS AND METHODS

### 2.1 Sample selection

This was a multicenter, retrospective cohort study at certified fertility clinics in Japan (Japanese Institution for Standardizing Assisted Reproductive Technology: JISART). We asked 11 ART facilities participating in JISART for clinical information about all cases without submucosal uterine myoma or hydrosalpinx, who received ERA between January 2018 and December 2020.

Inclusion criteria for analysis were that (1) ERA was conducted during a hormone replacement cycle (HRC), (2) Subsequent personalized blastocyst ET was conducted under HRC after ERA. All participating ART facilities offered ERA testing for patients with RIF.

### 2.2 Patient characteristics

Patient characteristics including age at the time of both oocyte retrieval and ET, body mass index (BMI), smoking status, infertility duration, pregnancy and delivery history were recorded. Additional clinical information including serum anti‐Müllerian hormone (AMH) level, infertility diagnoses, and numbers of previous ETs were also assessed.

### 2.3 Endometrial sampling for ERA

From Day 3 of the menstrual cycle, a transdermal estradiol patch (Estrana Tape 0.72 mg, Hisamitsu Co., Japan) was used for 10–14 days, and oral conjugated estrogen tablets (Premarin, 0.625 mg, Pfizer Japan Inc., Japan) were added if necessary. If endometrium thickness was ≥7 mm with an adequate serum estradiol level, either 300‐mg vaginal progesterone suppositories (Lutinus, Ferring pharmaceuticals, Japan) three times daily or chlormadinone acetate (Fuji Pharma, Japan) (12 mg per day) were initiated. If serum progesterone levels were >1 ng/mL at the time of progesterone initiation, that cycle was canceled. Endometrial biopsy was performed from the uterine fundus using a Pipet Curet (Endosuction, Hakko Company, Ltd., Japan) as described earlier.^8^ From the time of progesterone initiation to the biopsy of endometrial sample, 8 facilities took endometrial samples 120 h after progesterone initiation (*n* = 625), while one facility took them at 108 h (*n* = 74), another at 118 h (*n* = 11) and a third facility took them at 123 h (*n* = 151). Biopsied endometrial samples were put into cryotubes containing 1.5 mL RNA and shaken for a few seconds. Samples were kept at 4°C for 4 h and shipped at room temperature to Igenomix Japan for ERA analysis. No patients received ERA testing multiple times during the study period. Further, there were no potential conflicts of interest between those conducting this research and the company performing ERA testing.

ERA test results enabled classification of each patient's WOI as “receptive” or “non‐receptive.” Non‐receptive patients were further classified as “pre‐receptive,” “early‐receptive,” “late receptive,” or “post‐receptive,” meaning that the status at the time of the biopsy was 24 h earlier, 12 h earlier, 12 h later, or 24 h later than the patient's individual WOI, respectively. Therefore, in a subsequent cycle, personalized vitrified‐warmed blastocyst transfer ET was performed according to individual patient timing for WOI, based on results of the ERA test, at the time that the result was non‐receptive.

### 2.4 Clinical outcomes

We investigated clinical outcomes of clinical pregnancies, miscarriages, ongoing pregnancies, and live births. Clinical pregnancy was defined as confirmation of a gestational sac in utero. Miscarriage was defined as complete expulsion of products of conception before 22 weeks of gestation. Ongoing pregnancy was defined as a pregnancy that continued beyond 12 weeks of gestation.

### 2.5 Statistical analysis

First, we evaluated whether patient characteristics, including clinical information for personalized ET, differed between receptive and non‐receptive groups for ERA testing, using Chi‐squared or Student's *t*‐tests. Second, clinical outcomes of the first personalized ET following ERA were evaluated in all patients (*n* = 861), patients with RIF, defined as patients without pregnancy after two ETs (*n* = 627), and ETs receiving preimplantation genetic testing for aneuploidy (PGT‐A) (*n* = 69), using a Chi‐squared test. Third, to evaluate factors associated with receptivity, we conducted multiple logistic regression and calculated odds ratios (ORs) and 95% confidence intervals (95% CIs) for receptivity. Variable selection for multivariable analysis was based on forward stepwise variable selection methods, considering effects of confounding factors. Finally, in order to evaluate associations between receptive status and clinical outcomes after ERA testing, we also investigated crude and adjusted ORs of receptive status for clinical outcomes among patients with RIF. Variables that demonstrated significant associations with receptivity, as well as treatment factors that demonstrated significant differences between receptive status, that is, assisted hatching, numbers of embryos transferred, and endometrial thickness at ET, were included in the adjusted analysis. All analyses were conducted using the Stata MP statistical package, version 17.0 (StataCorp LLC, College Station, TX, USA). A two‐tailed *p*‐value of <0.05 was considered statistically significant.

## 3 RESULTS

**FIGURE 1:**
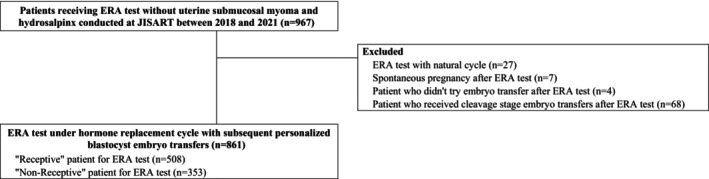
Flowchart.

A sample flow chart is shown in Figure 1. Data from 967 patients who received an ERA test, but who did not have uterine submucosal myoma or hydrosalpinx, were collected by participating ART facilities. Of these 967 patients, 27 with ERA tested under natural cycles were excluded, as were 7 with spontaneous conception after ERA testing, 4 who did not perform ETs after ERA testing, and 68 who received cleavage‐stage embryo transfers after ERA testing. The remaining 861 cases tested under HRC with subsequent personalized blastocyst ET information were included in the analysis.

Overall, 353 patients (41.0%) were non‐receptive (Table 1). Mean age at oocyte retrieval and ETs were both significantly higher in non‐receptive patients (*p* < 0.001). Non‐receptive patients were more likely to have been smokers and to have had longer infertility than receptive patients (*p* < 0.05). Proportions of infertility diagnoses and time from progesterone initiation to ERA testing were similar in both groups.

**TABLE 1:** Sample characteristics of infertile women who underwent ERA testing (*n* = 861)^a^.

Characteristics	Receptive (*n* = 508)	Non‐receptive (*n* = 353)	*p*‐Value
Patient age at oocyte retrieval (years)	36.5 ± 4.1	37.7 ± 3.8	<0.001
Patient age at embryo transfer (years)	37.2 ± 4.0	38.5 ± 3.9	<0.001
<30	23 (4.5%)	4 (1.1%)	<0.001
30–34	102 (20.1%)	61 (17.3%)	
35–39	216 (42.5%)	133 (37.7%)	
40–42	139 (27.4%)	110 (31.2%)	
≧43	28 (5.5%)	45 (12.8%)	
Serum AMH level (ng/mL)	3.1 ± 2.7	2.9 ± 2.8	0.40
BMI (kg/m^2^)	21.4 ± 3.0	21.7 ± 3.2	0.21
Smoking	26 (5.1%)	52 (14.7%)	<0.001
History of clinical pregnancy	157 (30.9%)	128 (36.3%)	0.10
Parous	61 (12.0%)	30 (8.5%)	0.10
Duration of infertility (months) (*n* = 859)	50.0 ± 38.4	65.2 ± 42.9	<0.001
Infertility diagnosis			
Male factor	138 (27.2%)	107 (30.3%)	0.31
Tubal factor	93 (18.3%)	63 (17.9%)	0.86
Edometriosis	54 (10.6%)	44 (12.5%)	0.40
PCOS/ovulation disorder	51 (10.0%)	27 (7.7%)	0.23
Poor ovarian reserve	25 (4.9%)	7 (2.0%)	0.06
Unexplained	120 (23.6%)	73 (20.7%)	0.31
Median number of previous ETs (*n* = 832)^b^	2 (2–3)	2 (1–3)	0.09
0	58 (11.4%)	66 (18.7%)	0.04
1	47 (9.3%)	34 (9.6%)	
2	168 (33.1%)	111 (31.4%)	
≧3	212 (41.7%)	136 (38.5%)	
Missing	23 (4.5%)	6 (1.7%)	
Time from progesterone initiation (hours)	119.4 ± 4.1	119.6 ± 3.1	0.58

Abbreviations: AMH, Anti‐Müllerian Hormone; BMI, body mass index; ERA, endometrial receptivity analysis; ET, embryo transfer; PCOS, polycystic ovary syndrome.

^a^ Data are presented as mean ± SD for continuous variables and *n* (%) for dichotomous variables unless otherwise specified.

^b^ Median (interquartile range).

**FIGURE 2:**
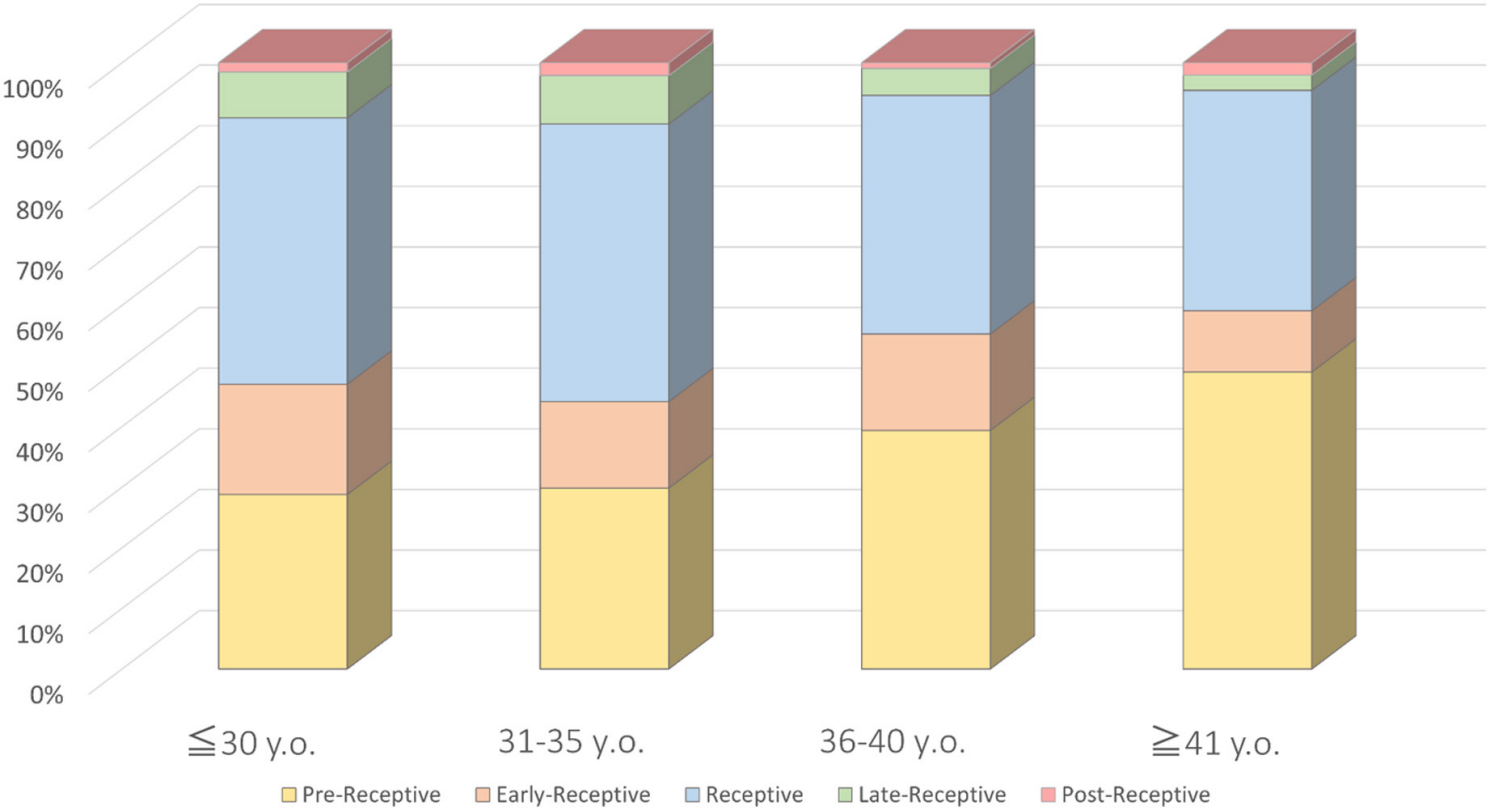
Proportions based on age at ERA testing.

Proportions of pre‐receptive patients increased with age: 21.3% (10/47) at age 30 or younger, 32.0% (62/194) at age 31–35, 39.4% (153/388) at age 36–40, and 49.1% (114/232) at age 41 or older, with a significant increasing trend (*p* < 0.01), while proportions of late receptive patients decreased from 6.4% (3/47) to 3.0% (7/232) (Figure 2). Accordingly, proportions of receptive patients decreased significantly with age.

Compared with patients with receptive status, non‐receptive patients were more likely to have received assisted hatching and single‐embryo transfer, while endometrial thickness at ET was significantly greater than in patients with receptive status (*p* < 0.05) (Table 2). Clinical pregnancy, miscarriage, ongoing pregnancy, and live birth rate among receptive patients were 44.5%, 26.1%, 32.9%, and 26.8%, respectively (Table 3), which were statistically similar to those of non‐receptive patients (43.1%, 28.3%, 30.9%, and 28.9%, respectively). Those results were unchanged in the RIF subgroup (*n* = 627) and ETs following PGT‐A (*n* = 69).

**TABLE 2:** Clinical characteristics of personalized blastocyst embryo transfers after ERA testing (*n* = 861)^a^.

	Receptive (*n* = 508)	Non‐receptive (*n* = 353)	*p*‐Value
Fertilization method			
IVF	155 (30.5%)	95 (26.9%)	0.25
ICSI	353 (69.5%)	258 (73.1%)	
ET following PGT‐A	43 (8.5%)	26 (7.4%)	0.56
Assisted hatching	442 (87.0%)	327 (92.6%)	0.009
ET using good morphological blastocyst	359 (70.7%)	243 (68.8%)	0.57
Single ET	440 (86.6%)	329 (93.2%)	0.002
Double ET	68 (13.4%)	24 (6.8%)	
Endometrial thickness at ET (mm) (*n* = 822)	10.2 ± 2.0	10.5 ± 2.5	0.045

Abbreviations: ERA, endometrial receptivity analysis; ET, embryo transfer; ICSI, intracytoplasmic sperm injection; IVF, in vitro fertilization; PGT‐A, preimplantation genetic testing for aneuploidy.

^a^ Data are presented as mean ± SD for continuous variables and *n* (%) for dichotomous variables unless otherwise specified.

**TABLE 3:** Clinical outcomes of personalized blastocyst embryo transfers following ERA testing.

Characteristics	Receptive	Non‐receptive	*p*‐Value
Overall (*n* = 861)			
Clinical pregnancy rate	44.5% (226/508)	43.1% (152/353)	0.68
Miscarriage rate	26.1% (59/226)	28.3% (43/152)	0.64
Ongoing pregnancy rate	32.9% (167/508)	30.9% (109/353)	0.54
Live birth rate	26.8% (136/508)	28.9% (102/353)	0.49
RIF (*n* = 627)			
Clinical pregnancy rate	43.4% (165/380)	41.7% (103/247)	0.67
Miscarriage rate	27.3% (45/165)	24.3% (25/103)	0.59
Ongoing pregnancy rate	31.6% (120/380)	31.6% (78/247)	1.00
Live birth rate	25.3% (96/380)	29.6% (73/247)	0.24
ET following PGT‐A (*n* = 69)			
Clinical pregnancy rate	65.1% (28/43)	61.5% (16/26)	0.76
Miscarriage rate	7.1% (2/28)	18.8% (3/16)	0.24
Ongoing pregnancy rate	60.5% (26/43)	50.0% (13/26)	0.40
Live birth rate	44.2% (19/43)	42.3% (11/26)	0.88

Abbreviations: ERA, endometrial receptivity analysis; ET, embryo transfer; PGT‐A, preimplantation genetic testing for aneuploidy; RIF, recurrent implantation failure.

In the crude analysis, older patients at ETs and those with longer duration of infertility had significantly lower odds for receptivity. In the multivariable model, significant associations between those variables and receptivity remained (Table 4). Further, a history of delivery offered significantly higher odds for receptivity, while smoking resulted in significantly lower odds. The number of previous ETs was not significantly associated with receptivity. After adjusting for potential confounders, there were no significant associations between receptive status in ERA and clinical outcomes (Table 5).

**TABLE 4:** Factors associated with endometrial receptivity.

	Crude OR (95% CI)	Adjusted OR (95% CI)
Patient age at embryo transfer		
<30 y.o.	Reference	Reference
30–34 y.o.	**0.29 (0.10 to 0.88)**	**0.32 (0.11 to 0.98)**
35–39 y.o.	**0.28 (0.10 to 0.83)**	0.35 (0.12 to 1.04)
40–42 y.o.	**0.22 (0.07 to 0.65)**	**0.27 (0.09 to 0.81)**
≧43	**0.11 (0.03 to 0.35)**	**0.19 (0.06 to 0.61)**
Serum AMH level (ng/mL)^a^	1.02 (0.97 to 1.10)	—
BMI (kg/m^2^)^a^	0.97 (0.93 to 1.02)	—
Smoking	0.79 (0.59 to 1.05)	**0.42 (0.25 to 0.70)**
History of clinical pregnancy	0.79 (0.59 to 1.05)	0.74 (0.52 to 1.04)
Parous	1.47 (0.93 to 2.33)	**2.05 (1.19 to 3.51)**
Duration of infertility (months)^a^	**0.99 (0.987 to 0.994)**	**0.99 (0.989 to 0.997)**
Infertility diagnosis^b^		
Male factor	0.86 (0.64 to 1.16)	—
Tubal factor	1.03 (0.72 to 1.47)	—
Endometriosis	0.84 (0.55 to 1.28)	—
PCOS/ovulation disorder	1.35 (0.83 to 2.19)	—
Poor ovarian reserve	**2.56 (1.09 to 5.98)**	—
Unexplained	1.19 (0.85 to 1.65)	—
Number of previous ETs		
0	**0.58 (0.38 to 0.89)**	—
1	**0.91 (0.55 to 1.51)**	—
2	Reference	—
≧3	1.03 (0.75 to 1.42)	—
Time from progesterone initiation (hours)^a^	0.99 (0.95 to 1.03)	—

*Note*: Bold value indicates significant *p* value < 0.05.

Abbreviations: AMH, Anti‐Müllerian Hormone; BMI, body mass index; CI, confidence interval; ET, embryo transfer; OR, odds ratio; PCOS, polycystic ovary syndrome.

^a^ ORs for one‐unit increase.

^b^ OR for those without factors.

**TABLE 5:** Crude and adjusted odds ratios of receptive status for clinical outcomes in personalized blastocyst embryo transfers following ERA testing among patients with recurrent implantation failures^a^.

Clinical outcomes	Crude OR (95% CI)	Adjusted OR (95% CI)^b^
Clinical pregnancy	1.07	1.00
	(0.78 to 1.48)	(0.70 to 1.43)
Miscarriage	1.17	1.70
	(0.66 to 2.06)	(0.87 to 3.35)
Ongoing pregnancy	1.00	0.86
	(0.71 to 1.41)	(0.59 to 1.25)
Live birth	0.81	0.74
	(0.56 to 1.15)	(0.50 to 1.09)

Abbreviations: CI, confidence interval; ERA, endometrial receptivity analysis; OR, odds ratio.

^a^ References are non‐receptive results for ERA testing.

^b^ Adjusted for patient age at embryo transfer, smoking status, history of delivery, duration of infertility, assisted hatching, number of embryos transferred and endometrial thickness at embryo transfer.

## 4 DISCUSSION

In this multicenter, cross‐sectional study at certified fertility clinics in Japan, 41.0% of patients (353/861) were non‐receptive. Clinical pregnancy, miscarriage, ongoing pregnancy, and live birth rates for first personalized blastocyst ET after ERA testing were similar between receptive and non‐receptive patients. Multiple logistic regression demonstrated that advanced age, smoking, and longer duration of infertility were negatively associated with receptivity, whereas a history of delivery was positively associated. Among patients with RIF, there were no significant associations between receptivity and clinical outcomes. To the best of our knowledge, this is the largest study to investigate clinical outcomes after ERA, as well as factors associated with endometrial receptivity.

Clinical pregnancy, miscarriage, ongoing and live birth rates for the first personalized ET were comparable for receptive and non‐receptive patients (Table 3). Those associations were unchanged after adjusting for confounders and excluding patients with RIF (Table 5). However, these results should be interpreted with caution. Clinical characteristics of personalized ETs between receptive and non‐receptive are different. In particular, the rate of single‐embryo transfers and assisted hatching were statistically higher in the non‐receptive group (Table 3). The reason for the low rate of single‐embryo transfer in the receptive group is that the receptive group experienced significantly higher numbers of previous ETs compared with the non‐receptive group (Table 1). Therefore, more patients requested double‐embryo transfers in expectation of a higher implantation rate. Similarly, the non‐receptive group had a higher rate of assisted hatching than the receptive group, probably due to the higher mean age of the non‐receptive group, in the hope of improving the implantation rate. The non‐receptive group indicated higher mean age at oocyte retrieval. Magli et al. have shown the effectiveness of assisted hatching for women of more advanced age.^15^ Therefore, the rate of assisted hatching implementation is expected to be higher in the non‐receptive group than in the receptive group, in an attempt to improve the implantation rate.

Because we did not compare the outcomes between patients with or without ERA testing, whether ERA has a positive impact on ART success is unknown. Previous reports on ERA demonstrated controversial results for the effect of ERA.^16^ Cozzolino et al. recently completed a retrospective multicenter cohort study.^17^ They reported clinical outcomes of personalized ET based on ERA results after a single, previous, failed transfer among autologous ETs (*n* = 255) and donor transfers (*n* = 319). They found that implantation, pregnancy, and clinical pregnancy rates were lower in patients undergoing personalized ET than ETs unguided by ERA. On the other hand, Liu et al. reported meta‐analyses, including observational studies about ERA testing efficacy. They reported that the ongoing pregnancy rate/live birth rate for those who followed personalized ET with ERA was somewhat higher (but nonsignificant) than for patients undergoing routine ET without ERA testing, among infertile patients with good prognoses (53.7% vs. 39.5%, OR = 1.28, 95% CI, 0.92 to 1.77). Further, among patients with RIF, the ongoing pregnancy rate/live birth rate for personalized ET among those who were non‐receptive, increased to the level of receptive patients undergoing ETs (40.7% vs. 49.6%, OR = 0.94, 95% CI, 0.70 to 1.26).^18^ Though we obtained similar results (Tables 3 and 5), observational studies cannot prove that use of ERA improves the success rate for ART. Thus, proper, large‐scale, randomized, control trials (RCTs) and meta‐analyses of those results need to be conducted to evaluate the utility of ERA testing.

Although our analysis did not show significant associations between the number of previous ETs and receptivity in either crude or adjusted analysis, several studies suggested a link between the number of implantation failures and proportions of receptive and non‐receptive patients. Mahajan et al. reported that 186 patients were divided into two groups based on the number of implantation failures. Among Group I persons who failed only one IVF cycle, 15.1% were non‐receptive, while among Group II patients, who failed three IVF cycles, 27.5% were non‐receptive (*p* = 0.04).^19^ On the other hand, Doyle et al. investigated 307 patients with ERA‐timed, single‐euploid FETs and found that there was no significant difference in the proportions of receptive and non‐receptive among those who previously failed FETs.^20^ They commented that patients with more previously failed euploid FET cycles are not at increased risk of a displaced WOI. Although several studies have analyzed numbers of repeated implantation failures, endometrial P4 and 17α‐hydroxyprogesterone expression, and chronic endometritis as factors that may influence receptivity, not all studies drew the same conclusions.^14^, ^19^, ^21^

Notably, our results demonstrated a significant increasing trend in the proportion of non‐receptivity, especially pre‐receptive, with increasing age, based on ERA testing (Figure 2). Possible reasons for this association include age‐related abnormalities of the endometrium and changes in progesterone receptor sensitivity. Eghlidi et al. reported that decreased expression of progesterone receptors and decreased ability of progesterone to interact with its receptors in hypothalami of rhesus macaques^22^ are associated with age. Further, although methods of progesterone administration and measurement of progesterone levels are controversial, effects of progesterone receptor activation on WOI have been previously reported,^10^ and progesterone receptor activation is a major driver in determining WOI and pregnancy.^23^ Based on these results, age‐related increasing proportions of pre‐receptive patients observed in our study may be mediated by diminished progesterone production and expression of its receptors.

Further, our study demonstrated that smoking is associated with significantly decreased receptivity. To date, no other studies have found a correlation between receptivity and smoking, based on ERA testing. Smoking is widely reported to have various negative effects on pregnancy.^24^, ^25^, ^26^, ^27^ In particular, effects on events from fertilization to implantation, such as decreased fertilization capacity and delayed blastocyst formation, have been reported.^28^ In addition, smoking is also negatively associated with maintaining pregnancy. Previous studies have demonstrated that smoking decreases female fecundity and increases the risk of spontaneous abortion as well as ectopic pregnancy.^29^, ^30^

Our results also demonstrate that the longer the duration of infertility, the more likely women are to be non‐receptive. Further, our analysis demonstrated a positive association between history of delivery and receptive results, suggesting that displaced WOI contributes to infertility. To the best of our knowledge, no other report has mentioned this association. Patients with WOI displacement are those who do not become pregnant after multiple attempts, which may support the present results that the duration of infertility and WOI displacement are positively correlated. Thus, for patients with longer infertility duration, it may be important to adjust implantation timing into the receptive range to achieve live births, as well as conception.

The strength of this study is that it comprised the largest investigation to date of those receiving ERA tests from multiple, certified ART facilities in Japan. The large sample enabled us to investigate effects of patient characteristics and clinical information on receptivity status using multivariable analysis. However, the study has several limitations. First, although all participating clinics offer ERA tests for patients with RIF, there are patients who have never failed ET or have failed only once (Table 1). This small heterogeneity in the sample could potentially introduce sampling bias. Second, the current study included a large sample of women receiving ERA from multiple certified ART facilities. Regimens for HRC varied among participating clinics, although there are no existing studies suggesting that regimens for HRC might affect receptivity. Similarly, though there were no significant associations between the time of progesterone initiation to biopsy and receptivity status, several facilities took endometrial samples at intervals other than 120 h, which may have affected study results. Third, we analyzed various patient characteristics, but there are other factors that could potentially affect receptivity, such as chronic endometritis and its treatment history, or endometrial polyps.^14^ Thus, a prospective study assessing those factors as well as clinical outcomes according to a uniform standard and HRC regimen is warranted.

In conclusion, using the largest sample to date, this study demonstrated that clinical pregnancy, miscarriage, ongoing pregnancy, and live birth rates for first personalized blastocyst ET after ERA testing were similar between receptive and non‐receptive patients. Further, advanced patient age, smoking, and increasing duration of infertility are negatively associated with endometrial receptivity, whereas a history of delivery was positively associated. Although effectiveness of ERA testing in achieving live births is still a matter of debate, based on our results, there may be candidates who need ERA testing. For a proper evaluation of the effect of ERA testing, it is necessary to the extent possible, to unify patient background factors, such as age at oocyte retrieval, numbers of previous implantation failures and the grade of blastocyst to be transferred; thus, ideally a large‐scale, randomized, controlled trial may be able to eliminate these biases.

## CONFLICT OF INTEREST STATEMENT

Yutaka Osuga and Hiroaki Yoshida are Editorial Board members of Reproductive Medicine and Biology and co‐authors of this article. To minimize bias, they were excluded from all editorial decision‐making related to acceptance of this article for publication.

## HUMAN RIGHTS STATEMENT AND INFORMED CONSENT

All procedures were performed in accordance with ethical standards of the institutional committees on human experimentation (institutional and national) and the Helsinki Declaration of 1964 and its later amendments.

## ANIMAL STUDIES

This article does not contain any studies with animal subjects performed by any of the authors.

## APPROVAL BY ETHICS COMMITTEE

This study was approved by the ethics committee of Kyono ART clinic Takanawa, study reference number 4905–210906.
